# First-in-man application of a novel therapeutic cancer vaccine formulation with the capacity to induce multi-functional T cell responses in ovarian, breast and prostate cancer patients

**DOI:** 10.1186/1479-5876-10-156

**Published:** 2012-08-03

**Authors:** Neil L Berinstein, Mohan Karkada, Michael A Morse, John J Nemunaitis, Gurkamal Chatta, Howard Kaufman, Kunle Odunsi, Rita Nigam, Leeladhar Sammatur, Lisa D MacDonald, Genevieve M Weir, Marianne M Stanford, Marc Mansour

**Affiliations:** 1 Sunnybrook Health Sciences Center, Toronto, ON, Canada; 2 Immunovaccine Inc, Halifax, NS, Canada; 3 Department of Microbiology & Immunology, Dalhousie University, Halifax, NS, Canada; 4 Duke Comprehensive Cancer Center, Durham, NC, USA; 5 Mary Crowley Cancer Research Center, Dallas, TX, USA; 6 University of Pittsburgh Cancer Institute, Pittsburgh, PA, USA; 7 Rush University Medical Center, Chicago, IL, USA; 8 Roswell Park Cancer Institute, Buffalo, NY, USA

**Keywords:** Immunotherapy, Peptide, Montanide, DepoVax^TM^

## Abstract

**Background:**

DepoVax^TM^ is a novel non-emulsion depot-forming vaccine platform with the capacity to significantly enhance the immunogenicity of peptide cancer antigens. Naturally processed HLA-A2 restricted peptides presented by breast, ovarian and prostate cancer cells were used as antigens to create a therapeutic cancer vaccine, DPX-0907.

**Methods:**

A phase I clinical study was designed to examine the safety and immune activating potential of DPX-0907 in advanced stage breast, ovarian and prostate cancer patients. A total of 23 late stage cancer patients were recruited and were divided into two dose/volume cohorts in a three immunization protocol.

**Results:**

DPX-0907 was shown to be safe with injection site reactions being the most commonly reported adverse event. All breast cancer patients (3/3), most of ovarian (5/6) and one third of prostate (3/9) cancer patients exhibited detectable immune responses, resulting in a 61% immunological response rate. Immune responses were generally observed in patients with better disease control after their last prior treatment. Antigen-specific responses were detected in 73% of immune responders (44% of evaluable patients) after the first vaccination. In 83% of immune responders (50% of evaluable patients), peptide-specific T cell responses were detected at ≥2 time points post vaccination with 64% of the responders (39% of evaluable patients) showing evidence of immune persistence. Immune monitoring also demonstrated the generation of antigen-specific T cell memory with the ability to secrete multiple Type 1 cytokines.

**Conclusions:**

The novel DepoVax formulation promotes multifunctional effector memory responses to peptide-based tumor associated antigens. The data supports the capacity of DPX-0907 to elicit Type-1 biased immune responses, warranting further clinical development of the vaccine. This study underscores the importance of applying vaccines in clinical settings in which patients are more likely to be immune competent.

**Trial Registration:**

ClinicalTrials.gov NCT01095848

## Background

Cancer immunotherapy has demonstrated clinical benefit in recent clinical trials [1-4]; however, in order to achieve greater efficacy, several challenges must be addressed. These include, i) poor immunogenicity of the chosen peptides and/or vaccine platforms, ii) inappropriate functional polarity of induced responder T cells, iii) inefficient trafficking into or poor integrity of specific T effector cells within the tumor microenvironment, iv) presence of tumor induced immune regulatory cells and v) suboptimal patient selection and integration with standard of care treatments. We sought to develop a vaccine strategy to address many of these challenges.

Our novel cancer vaccine DPX-0907 contains a polynucleotide-based adjuvant and a universal T helper peptide, along with seven HLA-A2 restricted peptides derived from tumor-associated antigens. These antigens are involved in multiple, critical cancer pathways such as tissue invasion and metastasis (P5; Integrin β8 subunit precursor, P14; Junction plakoglobin and P15; EDDR1), evading apoptotic cell death (P3; BAP31) and providing the ability to resist anti-growth signals (P7; Abl binding protein C3) [5-9], with resultant specific immune responses expected to reduce the chance for progression of tumor escape variants [9,10]. These peptides were among 16 described using mass spectrometry analysis of HLA-A2-bound peptides from HLA-A2^+^ ovarian cancer cell lines [5]. We have previously described and tested this vaccine candidate in preclinical models [5,11]. The vaccine-incorporated peptides are presented by MHC class I on the cell surface of breast, ovarian and prostate cancer cells, but not on normal cells [6]. Their inclusion in DPX-0907 yields an immunogenic vaccine in HLA-A2 transgenic mice that promotes the activation of both Type 1 T cell responses, while minimizing the induction of regulatory mechanisms [11].

In order to enhance the potency of a peptide platform, we developed a novel vaccine platform called DepoVax^TM^[11], a liposome-in-oil platform containing stable components that does not require creation of an emulsion, simplifying the use of oil-based depot vaccines in the clinic. DepoVax can be custom-formulated with mixtures of CD8^+^ T cell peptide epitopes, a T helper epitope derived from tetanus toxoid [12], and an adjuvant of choice. The liposomes carry incorporated hydrophilic antigens and adjuvant directly into an oil medium such as Montanide ISA51 VG, entrapping all vaccine ingredients in a form amenable for efficient uptake and processing/presentation by antigen presenting cells (APCs). DepoVax-formulated vaccines can induce effective immune responses after single dose administration [11,13-15]. In the current report, we describe the safety and immunogenicity of a phase I trial of DPX-0907 in HLA-A2^+^ patients with advanced breast, ovarian and prostate cancer.

## Materials and methods

### Patient population and trial design

Specific eligibility criteria for each type of cancer were: i) ovarian cancer: patients with stage III or IV ovarian cancer with evidence of a complete or partial response by radiological imaging after front-line debulking surgery and platin-based cytotoxic therapy or patients with metastatic ovarian cancer, clinically or radiologically stable disease for greater than 3 months after completion of first-line therapy; ii) breast cancer: patients with stage IV breast cancer who had received at least one course of hormonal or cytotoxic therapy for metastatic cancer, had completed their course of cytotoxic therapy and had been off therapy with stable disease or better for ≥3 months. Continued hormonal therapy was permitted; iii) prostate cancer: patients with rising PSAs or increases in measurable disease after at least one course of an accepted hormonal therapy and castrate testosterone levels (<50 ng/dL) or who had received previous courses of cytotoxic chemotherapy. Where applicable, patients with prostate cancer remained on anti-androgen therapy during the trial. All patients were positive for the HLA-A2 haplotype and met other standard inclusion /exclusion criteria with a life expectancy of at least 6 months.

Subjects received three subcutaneous injections of the DPX-0907 vaccine three weeks apart in the same upper thigh region, either at 0.25 mL (dose A) or 1.0 mL (dose B), according to the schedule shown in Additional file 1: Figure S1. The study was conducted in accordance with ethical guidelines of the Declaration of Helsinki. The protocol and patient-informed consent form received approval by individual Institutional Review Boards and ethics committees. Written informed consent was obtained for all patients.

### Vaccine formulation

DPX-0907 contained seven MHC class I-presented peptides (P4, P5, P7, P13, P14, P15 and P3 corresponding to peptides from Topoisomerase II α, Integrin β8 subunit precursor, Abl-binding protein C3, TACE/ADAM 17, Junction plakoglobin, EDDR1 and BAP31 respectively) which were isolated from HLA-A2^+^ ovarian cancer cell line (Additional file 2: Table S1) [5]. The vaccine containing these synthetic peptides (Polypeptide Inc., formerly NeoMPS, San Diego, CA) and a T helper peptide epitope (modified tetanus toxin peptide, 830-844; AQYIKANSKFIGITEL; A16L) was formulated in a proprietary DepoVax formulation as described in published work [11]. The aqueous liposomal solution was sized to 120 nm particle size through extrusion, lyophilized and shipped to clinical sites along with a vial of Montanide ISA51 VG (SEPPIC, France) and the clinical kits were stored at 4°C until use. Just before use, the lyophilized vaccine cake was reconstituted in Montanide ISA51 VG for injection.

### Immune monitoring

DPX-0907-induced immune responses in the peripheral blood of vaccinated patients were investigated at baseline (SD0) and following each of the three doses administered (SD21, three weeks after first dose; SD42, three weeks after second dose; SD73, a month after third and final vaccination). Peripheral blood mononuclear cells (PBMC) were frozen in liquid nitrogen tanks and were shipped in batches to National Immune Monitoring Laboratory or NIML, (Montreal, QC) for immunological assessment.

#### Pentamer staining

The following Pro5® MHC-pentamers conjugated to PE were purchased from ProImmune (Oxford, UK): A*0201–NLVPMVATV (CMV), A*0201–SLYNTVATL (HIV), A*0201–FLYDDNQRV (P4), A*0201-YLIELIDRV (P13), A*0201–NLMEQPIKV (P14), A*0201–FLAEDALNTV (P15), A*0201–ALMEQQHYV (P5), A*0201–ILDDIGHGV (P7) and A*0201-KLDVGNAEV (P3). PBMC from patients and healthy donors were thawed and rested overnight (12-18 h) in complete RPMI media and were analyzed directly (ex vivo) or after in vitro activation for 10 days in the presence of individual peptides from the vaccine (10 μg/mL from day 0-3 and 5 μg/mL from day 4-10) and IL-2 (10 IU/mL), IL-15 (10 ng/mL). The pentamer analysis consisted of staining the PBMC with pentamer at 4°C, followed by a wash step, and staining for the surface markers CD3, CD8 and CD45RA in combination with a cell viability marker. This assay was validated before patient samples were analyzed and inter- and intra-assay variations were strictly controlled using the positive control CMV-pentamer and negative control HIV-pentamer. The CD3^+^ T cells were selected from total live gated cells and were further separated into CD4 and CD8 T cells. The CD8 T cells were further analyzed based on CD45RA expression and pentamer positivity to discriminate between memory and naïve population. More than two-fold higher frequencies of pentamer-positive CD8 T cells compared to pre-treatment baseline values were considered as positive responders to vaccine treatment. In addition, a staining frequency value of greater than two standard deviations above background staining by negative control HIV-pentamer (range 0.00-0.03%) or CD8 negative cells (range 0.02-0.06%) were required to consider a sample a positive response. The limit of detection for this assay is 0.02% frequency of pentamer-specific cells.

#### Intracellular cytokine staining

Briefly, 10^6^ PBMC, after overnight resting, were stimulated for 1 hour with individual peptides and pools of peptides (with and without A16L) in the presence of anti-CD107a antibodies. Final A16L peptide concentration was 0.5 μg/mL and stimulations with peptide pool were done at 1 μg/mL (for each peptide). Experimental controls included unstimulated PBMC, PMA/Ionomycin and CEF (CMV/EBV/FLU) peptide pool stimulated PBMC. Protein secretion inhibitors (GolgiPlug™/GolgiStop™, BD Bioscience) were added after 1 hour of stimulation, and cells were incubated for an additional 5 hours at 37°C and 5% CO_2_. Following in vitro stimulation, cells were washed and surface stained (CD8, CD27 CD3, CD4, CD45RA, CD107a and viability marker) followed by intracellular staining (IFN-γ, TNF-α, IL-17, IL-2 and IL-4) of fixed/permeabilized cells. For gating, a standard method was used where live cells were gated for CD3 positive T cells, and were then gated further to separate CD4 and CD8 T cells. The CD27 and CD45RA markers were then used, and cells phenotyped as naïve, central memory, effector memory and late differentiated T cells. Each of these populations was tested for intracellular cytokine production following peptide stimulation.

Labeled cells were acquired on a LSR II flow cytometer using the FACS DiVa software (BD Bioscience) and analyzed using FlowJo software. Multifunctional cytokine analysis was performed after stringent gating of each cytokine positive population. In addition, SPICE, a data mining software application, was used to analyze large FlowJo data sets from polychromatic flow cytometry and to organize the normalized data graphically. A positive cytokine response was defined as more than two-fold increase in the frequency of cytokine secreting cells following peptide-stimulation compared to corresponding non-stimulated cells. Furthermore, vaccine-induced changes were considered positive when such cytokine positive cell frequency was more than two-fold compared to pre-treatment samples and showed ≥ 0.05% frequency. The limit of detection of this assay is 0.01% of responder cells.

#### IFN-γ ELISpot

IFN-γ ELISpot was performed at Cellular Technology Limited (Shaker Heights, OH). Patient PBMC or non-vaccinated healthy control PBMC were plated at 3x10^5^ cells per well in 96 well ELISpot plates. Cells were left unstimulated or stimulated with a range of indicated individual peptides (10-100 μg/mL) or with similar concentrations of the pooled DPX-0907 peptides for 24 h. The optimum concentration of peptide for stimulation in this assay was 25-50 μg/mL. PHA was used as a positive control stimulus. Antigen-specific induction of IFN-γ was measured by capturing the cytokine with plate-bound antibodies followed by developing and counting the number of spot forming units (SFU) in each well using automated plate scanner. The limit of detection of this assay is 0.01% of cells that secrete cytokines upon stimulation.

## Results

### Patient baseline characteristics

A total of 23 cancer patients qualified to participate in the study based on inclusion criteria, and included 13 prostate cancer, 7 ovarian cancer and 3 breast cancer patients. One of the ovarian cancer patients in the dose B group discontinued the study due to an unrelated serious adverse event (SAE) following a single vaccination, leaving a total of 22 patients with 11 patients in each of the dosage groups. Details on patient’ age, sex, race, history and number of previous treatments for all patients enrolled are summarized in Additional file 2: Table S2. The prostate cancer patients had a median age of 65 years (range 54-76 years) while breast cancer patients had a median age of 46 (range 44-61 years) and ovarian cancer patients had a median age of 49 years (range 48 to 67 years). Of note, most of the patients (16/23, 70%) had a history of ≥3 anti-cancer treatments (cytotoxic/hormonal/radiation) and 4 patients (including one patient who did not complete all three vaccinations) had received a previous immunotherapy (Avastin/Provenge, Table 1).

**Table 1:** Age, disease status, stage, response to last therapy and clinical outcome for subjects receiving a complete set of three injections

**Subject ID**	**Age (years)**	**Treatment history**	**Stage**	**Response to Prior Treatment**	**Dose (mL)**	**Immune Response**	Time to Progression* (months)
**Breast Cancer**							
01-13	44	cytotoxic therapy, hormonal therapy, immunotherapy (Avastin), radiation	IV	SD	0.25	Yes	2 ^a^
04-06	61	concurrent hormonal therapy	IV	SD	0.25	Yes	> 8
04-19	46	cytotoxic therapy, concurrent hormonal therapy, radiation	IV	SD	1.0	Yes	2.5 ^a^
**Ovarian Cancer**							
05-14	50	platinin-based cytotoxic therapy	III	CR	0.25	Yes	4 ^a^
05-15	48	platinin-based cytotoxic therapy	III	CR	0.25	Yes	5 ^a^
02-01	49	platinin-based cytotoxic therapy	III	CR	0.25	No	> 9
05-07	54	platinin-based cytotoxic therapy	III	CR	1.0	Yes	> 8
02-09	67	platinin-based cytotoxic therapy, immunotherapy (Avastin)	IV	CR	1.0	Yes	> 8.5
01-22	49	platinin-based cytotoxic therapy	III	CR	1.0	Yes	> 8
**Prostate Cancer**							
03-17	60	hormonal therapy, anti-androgen therapy	Unknown	PD	0.25	Yes	2 ^a^
01-04	67	hormonal therapy, cytotoxic therapy, anti-androgen therapy, immunotherapy (Provenge), radiation	IV	PD	0.25	No	2 ^a^
02-05	63	ongoing hormonal therapy, cytotoxic therapy, concurrent anti-androgen therapy	IV	PD	0.25	Unknown	UE ^b^
03-16	65	hormonal therapy, cytotoxic therapy, anti-androgen therapy, radiation	IV	PD	0.25	No	2.3 ^a^
02-02	66	hormonal therapy, cytotoxic therapy, anti-androgen therapy, radiation	IV	PD	0.25	Unknown	5.7 ^a^
01-03	76	hormonal therapy, anti-androgen therapy, radiation	IV	SD	0.25	No	UE ^b^
02-12	54	hormonal therapy, concurrent anti-androgen therapy, radiation	IV	SD	1.0	Unknown	2.3 ^a^
01-18	68	hormonal therapy, anti-androgen therapy, radiation	IV	PD	1.0	Yes	2.3 ^a^
02-20	64	hormonal therapy, anti-androgen therapy, radiation	IV	Unknown	1.0	Unknown	2.3 ^a^
03-21	74	hormonal therapy, anti-androgen therapy, radiation	IV	PD	1.0	No	2.3 ^a^
04-10	63	hormonal therapy, concurrent anti-androgen therapy, radiation	IV	PD	1.0	Yes	3.5 ^c^
03-08	60	hormonal therapy, anti-androgen therapy, radiation	IV	PD	1.0	No	6.5 ^a^
02-11	68	hormonal therapy, anti-androgen therapy, radiation	IV	PD	1.0	No	7.3 ^a^

*CR* complete response, *SD* stable disease, *PD* progressive disease, *UE* unevaluable.

*since study day 0.

^a^Disease progression necessitating some other type of treatment.

^b^Withdrawal of consent.

^c^Death due to disease progression.

### Safety

Of the 22 patients who were vaccinated with the full cycle (three injections) of DPX-0907, no dose limiting toxicities (DLTs) were reported. A summary of the reported adverse reactions that are possibly, probably or definitely related to the different dose levels of DPX-0907 treatment is shown in Table 2. The most common adverse events were grade 1 and 2 injection site induration and erythema for both dose cohorts. A higher incidence of grade 2 injection site erythema, edema and pain were observed in patients receiving the 1.0 mL dose vaccine. Two grade 3 ulcerations and a single grade 3 cellulitis were reported after repeated immunizations with the 1.0 mL dose in the same vicinity. In one patient, this ulceration and cellulitis were reported 3 weeks after the third vaccination, which completely resolved by the next follow-up visit. In another patient, the ulceration was noted 6 weeks after the third vaccination, which improved to grade 1 by the next follow-up visit. At the 6 month follow up, most injection site reactions in patients had resolved to grade 1. There were no vaccine related grade 3 or greater adverse events after three immunizations with the 0.25 mL dose. Overall, four SAEs unrelated to DPX-0907 were observed including: i) abdominal pain due to disease progression resulting in hospitalization and eventually death, ii) pneumonia resulting in hospitalization, iii) partial bowel obstruction due to disease progression resulting in hospitalization, and iv) urinary obstruction due to disease progression resulting in hospitalization. No clinical signs of autoimmunity were seen in any treated patient.

**Table 2:** Safety and reported adverse events, occurring in two or more subjects, which were possibly, probably or definitely related to DPX-0907 treatment

**Toxicity**	**Total No. of Patients**		**0.25 mL Group**		**1 mL Group**		
	**0.25 mL**	**1 mL**	**Grade 1**	**Grade 2**	**Grade 1**	**Grade 2**	**Grade 3**
Injection site induration	11	9	6	5	3	5	1
Injection site erythema	8	10	6	2	5	4	1
Injection site pain	3	8	1	2	3	5	
Injection site pruritis	3	2	3		1	1	
Injection site warmth	2	2	1	1	1	1	
Injection site hematoma	2		2				
Injection site edema	1	4	1		2	2	
Injection site discoloration	1	2		1	2		
Injection site rash	1	1	1			1	
Injection site urticaria	1	1	1			1	
Injection site dry skin	1	1	1		1		
Injection site ulceration		3			1		2*
Pain	3	1	2	1		1	
Fatigue	2		1	1			
Fever	1	2		1	1	1	
Arthralgia	1	1	1		1		
Myalgia	1	1	1		1		
Anaemia	1	1		1		1	

*Appeared three or more weeks after of third injection and completely resolved.

### Clinical results

Since long-term monitoring was not included in the clinical plan, and only 5 of 22 patients completed the full 6 month follow up visit, the maximum time to progression period recorded in the study was 8-9 months starting from the day of a patients’ first vaccination (i.e. SD0). Hence, patients who reached the 6 month follow-up visit and remained disease free at that time were assigned a time to progression of >8 to 9 months. A summary of disease status, treatment history, response to earlier treatments, and time to progression is summarized in Table 1. During the study period 14 patients progressed, most of whom had prostate cancer (10 of 14 patients). In addition, one prostate cancer patient died of progressive disease 3.5 months into this study. Information on disease progression is not available for the remaining two prostate cancer patients since they opted out of the study. In contrast, disease progression was reported for 2 of 3 breast cancer patients and only 2 out of 6 ovarian cancer patients. Based on the favorable response to previous treatment, the breast cancer and ovarian cancer patient populations were less likely to be compromised immunologically and thus were evaluated together. Among patients who completed follow up, 1 of 3 breast cancer patients and 4 of 6 ovarian cancer patients had a time to progression of >8-9 months. These patients were still within the median progression free survival period for their previous treatment, thus a clinical benefit of vaccination cannot be ascertained in this study. Two of the five breast/ovarian cancer patients were treated in the dose A cohort and three were treated with the dose B vaccine.

### Immune monitoring

An important secondary objective of this trial was to determine the magnitude and diversity of cell-mediated immune responses induced by vaccination against the 7 cancer-associated peptide epitopes included in DPX-0907. This was measured primarily by MHC-pentamer staining for antigen-specific CD8^+^ T cells and multi-parametric flow cytometry using the intracellular cytokine staining (ICS) assay. An exploratory ELIspot assay was performed on select samples. Although a total of 22 patients received the full cycle of vaccinations, the quality of PBMC and the recovery of cells following thawing were poor in 4 prostate cancer patient samples, leaving samples from 18 patients that were deemed ‘evaluable’ for immune analysis.

Due to the relative short duration of the trial and the limited number of vaccine doses that could be applied to patients, an immune response was defined as a response to one or more vaccine antigens at one or more of the time points analyzed post-vaccination. Among the 18 patients evaluable for immune endpoints in this study, the highest frequency of positive immune responses was observed among the breast and ovarian cancer patients. All 3 (100%) breast cancer patients and 5 of 6 (83%) ovarian cancer patients responded to antigens included in the vaccine for a combined immune responder rate of 89%. Among prostate cancer patients, 3 of 9 (33%) showed a positive immune response to vaccination. Taken together, 11 of 18 patients (61%) showed measurable immune responses against one or more of the vaccine antigens (Table 3). While two patients’ response were measurable only by MHC-pentamer testing and 3 patients were positive only using the ICS assay, 6 of 11 patients’ responses were detectable by both the methods used. Immune responses were detectable as early as SD21 in 8 of 11 responders (73% or 44% of evaluable patients) suggesting that just one immunization was sufficient to elicit specific T cell responses in these patients. In 7 of 11 responders (64%, or 39%, of evaluable patients), specific response to cancer antigens was observed at SD73, suggesting the persistence of immune memory in peripheral blood. The 18 patients evaluable for immune response were equally split between dose cohorts (Table 4). Five of 9 0.25 mL (dose A) vaccine recipients (56%) and 6 of 9 patients (67%) treated with 1.0 mL (dose B) vaccine were categorized as immune responders.

**Table 3:** Immune outcome in DPX-0907-treated patients

		
Total patients recruited		23
Full treatment received		22
Evaluable		18
Immune response (IR)		
Positive IR in:	Breast cancer	3/3 (100%)
	Ovarian cancer	5/6 (83%)
	Breast/Ovarian	8/9 (89%)
	Prostate cancer	3/9 (33%)
	All patients	11/18 (61%)
	Dose A	5/9 (56%)
	Dose B	6/9 (67%)
Positive IR after:	1 vaccination	8/11 (73%)
	2 vaccinations	2/11 (18%)
	3 vaccinations	1/11 (9%)
Positive IR at:	1 time point	2/11 (18%)
	2 time points	7/11 (64%)
	3 time points	2/11 (18%)
Existing IR at:	SD73	7/11 (64%)

**Table 4:** The Dose A and B vaccine recipients who responded to DPX-0907 vaccination and their immune response to vaccine antigens

**Patient** **ID**	**Cancer Type**	**Vaccine Dosage**	**MHC Pentamer**		**ICS – Flow cytometry**		
			Antigen Positive	Study day Positive	Positive Stimulus	Study day Positive	Multi-cytokine Positive
04-06^a^	Breast	0.25 mL	P5 P15	21, 42 42	P5 Pool	42 42	Yes Yes
01-13^b^	Breast	0.25 mL	None	NA	Pool	21	Yes
05-14^b^	Ovarian	0.25 mL	P4	21, 42	Pool	21, 42^c^	Yes, No^c^
05-15^b^	Ovarian	0.25 mL	P3 P7 P13	73 73 73	None	NA	No
03-17^b^	Prostate	0.25 mL	None	NA	Pool	21^c^, 73	No^c^, Yes
04-19^b^	Breast	1.0 mL	None	NA	Pool	42, 73	Yes, Yes
05-07	Ovarian	1.0 mL	P15	73	Pool	21	Yes
02-09	Ovarian	1.0 mL	P5 P3	42, 73 42	None	NA	No
01-22^b^	Ovarian	1.0 mL	P5	21, 73	Pool	21, 42^c^, 73^c^	Yes, No^c^ No^c^
04-10^a^	Prostate	1.0 mL	P5 P7 P15	21, 42 42 21, 42	P13, P15, Pool	42 42^c^ 42	Yes No^c^ Yes
01-18^b^	Prostate	1.0 mL	P4 P7	42 21, 42, 73	Pool	21^c^, 42^c^, 73^c^	No^c^, No^c^, No^c^

^a^MHC-multimer positive CD8 T cells were also detected ex vivo in these patients: Subject 04-06, P5 (0.12% staining frequency) on SD42; Subject 04-10, P5 on SD21 (0.11%) and on SD42 (0.23%).

^b^PBMC from these patients were tested against pooled peptides only in ICS assay.

^c^T cells from PBMC at the indicated study day produced detectable levels of one of the cytokines tested (IFN-γ, TNF-α, IL-2).

MHC-pentamer reagents incorporating individual peptides contained in DPX-0907 were used to detect specific CD8^+^ T cell responses in patient blood ex vivo or after a 10 day stimulation of T cells in vitro with individual peptides. While most patients required in vitro stimulation of PBMC to expand and detect multimer positive cells, it was possible to detect such specific T cells ex vivo in two patients (Table 4). The frequency of ex vivo detected cells was further expanded following in vitro culture. Vaccination with DPX-0907 induced specific response against 6 of 7 tumor-associated peptides (all except P14; Table 4). The most frequent responses were seen against peptide P5, followed by P7 and P15. Five of 11 responding patients (45% or 28% of evaluable patients) were positive for responses to two or more peptides contained in DPX-0907.

**Figure 1:**
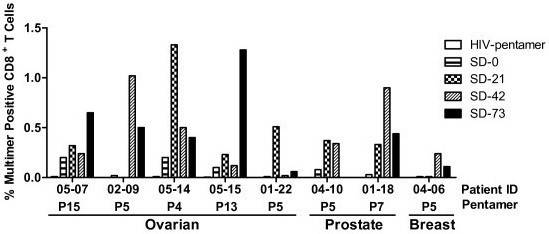
**The generation of antigen-specific CD8.**^**+**^**T cells in DPX-0907 vaccinated ovarian, prostate and breast cancer patients.** Patient PBMC were stimulated with indicated peptides in vitro as described in Materials and Methods. Cells were stained with corresponding MHC-peptide pentamer reagents to detect CD8^+^ T cells with peptide-specific TCR repertoire. HIV-pentamer served as a negative control (patients pre-screened for being HIV negative) and CMV-specific pentamer was used on a known CMV-positive donor PBMC as internal positive control for the assay (data not shown). Samples from all study time points were run simultaneously in the assay for each patient. Data represented as percentage of live gated CD3^+^CD8^+^ cells that were positive for pentamer staining. The background staining on CD8 negative cells (0.02-0.06%) has been subtracted from the values shown and the values for control HIV-pentamer staining were in the range of 0.00 to 0.03% for all patients.

Among 11 responders in this study, 8 patients (73% or 44% of evaluable patients) were positive in MHC-pentamer testing, with two-fold or higher increase in the frequency of specific CD8^+^T cells compared to baseline values. In some instances, the increase was >50-fold at post-vaccination time point compared to SD0 (Additional file 2: Table S3, data shown as fold-increase over baseline). Due to low level responses seen ex vivo, in vitro expansion using antigen peptides and cytokines was necessary in most patients; however, responses seen ex vivo were reproduced following in vitro activation. Some patients exhibited pre-existing immune responses (>two-fold higher than background control) and in some instances the vaccination stimulated expansion of these responses (Additional file 1: Figure S2). Sequential changes in the frequency of antigen-specific T cells at pre-vaccination baseline and at different post-vaccination study days are shown in Figure 1. Although the peak response occurred on different post-vaccination days for different responders, in almost all patients the frequency of such cells remained higher at SD73 compared to SD0. Results from three representative responders identified by the MHC-pentamer assay, with distinct CD8^+^pentamer^+^ double positive cells, are shown in Additional file 1: Figure S3, with the data representing actual percentage frequency of CD8^+^/CD45RA^-^/multimer^+^ (antigen-experienced memory) cells.

The ICS assay allows a broader analysis of functionally relevant antigen-specific T cells following vaccination. ICS assays were performed with pools of peptides in most patient samples (n = 10 for pooled peptides and n = 8 where individual peptides were tested along with the pool of peptides) due to limited numbers of available PBMCs. Multi-parametric flow cytometry was performed on patient PBMC to identify the type of responding T cells (Total CD8; central and effector memory CD8 cells) along with their ability to simultaneously secrete Th1 type protective cytokines, namely IFN-γ, TNF-α and IL-2. The data from cells/peptides from responding patients have been presented (Tables 4 and 5) and the data indicate T cells are producing multiple cytokines at the single cell level. Among eleven immune responders, nine (82%, or 50% of evaluable patients) were positive in the ICS assay with a two-fold or higher increase in the frequency of one or more cytokine secreting CD8^+^ T cells compared to baseline values (Table 5). Central memory CD8^+^ T cells (T_CM_, CD45RA^-^CD27^+^) were detected in these patients suggesting that the vaccine induced antigen-specific memory responses. Cytokine positive cells were more frequent in the T_CM_ compartment than among total CD8^+^ T cells (data not shown), in keeping with previous activation of T_CM_ cells. IFN-γ-secreting effector memory cells (T_EM,_ CD45RA^-^CD27^-^) were also detected occasionally (data not shown), but multi-functional T_EM_ cells were not a significant feature of the immune responses detected at the specified timepoints.

**Table 5:** The generation of multi-functional T cells in patients who responded to vaccination with DPX-0907

**Patient ID**	**Indication**	**Vaccine Dosage**	**Antigen- specific Intracellular cytokine staining**				
			**CD8**^**+**^**T cell population**	**Multi-cytokine positivity**	**Cytokines**		
					**IFNγ**	**TNFα**	**IL-2**
04-06^a^	Breast	0.25 mL	Total	Double	+	+	-
			Total	Triple	+	+	+
			T_CM_	Double	+	+	-
				Triple	+	+	+
01-13^a^	Breast	0.25 mL	Total	Double	+	+	-
			T_CM_	Double	+	+	-
05-14	Ovarian	0.25 mL	Total	Double	+	+	-
			Total	Triple	+	+	+
			T_CM_	Double	+	+	-
			T_CM_	Triple	+	+	+
03-17	Prostate	0.25 mL	Total	Double	+	+	-
04-19	Breast	1.0 mL	T_CM_	Double	+	+	-
			T_CM_	Triple	+	+	+
05-07	Ovarian	1.0 mL	Total	Double	+	+	-
			T_CM_	Double	+	+	-
01-22^a^	Ovarian	1.0 mL	Total	Double	-	+	+
04-10	Prostate	1.0 mL	Total	Double	+	+	-
			Total	Double	+	-	+
			Total	Triple	+	+	+
01-18^a^	Prostate	1.0 mL	Total	Single	+	-	-
			Total	Single	-	+	-
			Total	Single	-	-	+
			T_CM_	Single	+	-	-

^a^ These patients also showed the presence of IFN-γ secreting T_EM_ cells.

Multi-functional T cells expressing IL-2 along with IFN-γ and TNF-α (Table 5) were detected in some patients. Three representative cancer patients whose samples demonstrate increased cytokine secretion following peptide stimulation are shown in Additional file 1: Figure S2.

Multifunctional T cells, as determined by the secretion of more than one cytokine simultaneously, were demonstrated in nearly all responding patients within their total CD8^+^ and/or CD8^+^ T_CM_ cell compartments. Four patients were positive for triple cytokine secreting multi-functional CD8^+^ T cells following vaccine treatment. Vaccine dosage did not influence the induction of multi-functional T cells, but the effect of dose is difficult to directly ascertain with this small sample size. Two examples of patients showing multi-functional CD8^+^ T cells are shown in Additional file 1: Figure S4. The presence of triple positive cells, induced only after vaccination, suggests that DPX-0907 was responsible for inducing multi-functional, anti-tumor effector cells. In addition, other cytokines such as IL-17, IL-4 and degranulation marker CD107a were also tested in the ICS assay. While there were no differences seen in the expression of IL-4 and IL-17 in our patient population throughout the treatment period, at least four patients showed expression of CD107a in combination with IFN-γ, TNF-α or both (data not shown).

As part of the ICS assay, the response of CD4^+^ T cells to the T helper epitope (A16L) included in DPX-0907 was also examined. Seventeen of our evaluable patient samples were tested in total, with nine PBMC samples tested with A16L alone and PBMC from eight patients were tested with a pool of DPX-0907 peptides, with or without the inclusion of A16L. In 13 patients CD4^+^ T cell responses were detected by ICS, including multiple cytokine secretion and four patients were considered non-responders. Interestingly, two patients exhibited both pre- and post-vaccination responses to A16L while 11 patients had a CD4 response to A16L post-vaccination only. In addition, all nine patients who demonstrated positive cytokine responses in their CD8^+^ T cell compartment by ICS also displayed positive response to A16L in their CD4^+^ T cell compartment. In contrast, four patients showed CD4^+^ T cell responses to A16L without a concurrent CD8^+^ T cell cytokine response.

For most patients, there were insufficient numbers of PBMCs collected to conduct further confirmatory immune assays. However, for two patients that were considered immune responders by both pentamer and ICS assays, there were sufficient PBMC to also test immune responsiveness using an IFN-γ ELISpot assay. Although this assay was not a primary portion of the immune monitoring strategy, it afforded the opportunity to further confirm immune responses in these two patients. The ELISpot confirmed the responses seen in the ICS assays for both patients (Table 4, Additional file 1: Figure S5), in that peptides/pools that induced the production of multiple cytokines in the ICS assay also induced the production of IFN-γ by ELISpot. Interestingly, an immune response to P13 peptide was detected in the ELISpot assay for patient 04-06, but no such response was recorded in the MHC-pentamer assay. Conversely, an immune response to P4 peptide was detected by the MHC-pentamer assay for patient 05-14 and not detected by ELISpot. No non-specific IFN-γ immuno-spots were seen in non-vaccinated healthy controls PBMC in response to DPX-0907 antigens, even at 10-fold higher antigen concentrations.

## Discussion

The purpose of this study was to determine the safety and immunologic efficacy of DPX-0907, a peptide vaccine incorporating a novel carrier and adjuvant formulation. The delivery system, DepoVax, is a liposome-in-oil platform designed to efficiently entrap hydrophilic antigens and adjuvant directly in the oil medium Montanide ISA51 VG without the need for emulsification. DepoVax attracts APCs to the site of vaccination, facilitating antigen cross-presentation in MHC class I to CD8^+^ T cells [11]. As a peptide-based vaccine, DPX-0907 has the advantages of easy manufacture and a favorable safety profile [16]. The formulation itself has the additional advantages of long term stability, easy storage and an easy to use reconstitution method that does not require emulsification.

The primary endpoint of safety was achieved in this first-in-man study. Repeated administration of DPX-0907 did not result in any dose limiting toxicities or serious adverse events related to vaccine treatment. The most frequent side effects were grade 1-2 injection site erythema and induration, consistent with other vaccines that incorporate Montanide ISA51 VG as the carrier medium [17-19]. There was no relationship between immune responses and the grade or frequency of site of injection reactions. When 0.25 mL (dose A) is compared to other vaccines that incorporate antigens in Montanide ISA51 VG emulsion with a similar oil content, DPX-0907, not requiring emulsification, results in lower reports of pain and no reports of grade 3 induration and swelling [19]. However, we did note greater frequency of pain and edema, and the presence of grade 3 induration, erythema and transient ulceration in patients receiving multiple injections at the dose B (1.0 mL) of DPX-0907. Though the adverse events seen at the 1.0 mL dose were largely transient in nature, any further studies with DepoVax-formulated vaccines will likely contain no more than 0.5 mL of diluent.

The secondary endpoint was evaluation of antigen-specific immune responses to DPX-0907. DPX-0907 vaccination resulted in the activation of antigen-specific T cell responses in 61% of evaluable patients. There were no differences (as measured by the frequency, magnitude or quality of specific T cell responses) observed in patients entered on either of the dose tiers (data not shown), supporting the lack of a vaccine dose/immune response relationship as reported by others [20-22]. However, other studies have demonstrated dose dependent immune responses in patients vaccinated with peptide antigens [23,24]. This discrepancy may be related to the antigen, formulation, peptide/protein length (and subsequent requirement for antigen processing), adjuvant, vaccination regimen and patient population used in these studies. The immune responses in this study were rigorously documented with state of the art antigen-specific immune monitoring and consistent results were generally seen with different immune monitoring assays.

DPX-0907 generates rapid and sustained immune responses [11]. Many patients generated immune responses after the first vaccination and had detectable immune responses at two or more time points post DPX-0907 vaccination. Responses could also be detected for least one month after the completion of the vaccination cycle, consistent with our preclinical data. This data compares favorably with other clinical trials in similar patient populations and was particularly favorable for breast and ovarian cancer patients. Morse *et al*[6] recently reported a phase I cancer vaccine study in ovarian and breast cancer patients targeting 12 antigens using MHC class I-restricted peptides, including the seven peptides used in DPX-0907 [5,6]. In that study, the peptides were formulated in a highly adjuvanted GM-CSF/Montanide ISA51 VG emulsion. The study demonstrated a 64% (9 of 14 patients) T cell response rate by ELISpot, after 3-10 injections of vaccine [6]. A similar level of CD8^+^ T cell responses was seen in patients with epithelial ovarian cancer using NY-ESO1 peptides or a mix of tumor associated antigen peptides, also following at least 2-5 vaccinations [25-27]. DPX-0907 induced a similar immune response rate with one vaccination (56%) and a higher immune response rate after three vaccinations (89%) in similar patient populations. It is not clear in the vaccine trials mentioned above if these vaccines could generate immune responses after one vaccination and multi-functional T cell responses were not documented.

“Multi-functional” T cells, secreting a diverse array of cytokines in response to cognate antigen, have been increasingly associated with host protection in preclinical vaccine and tumor immunotherapy models and in HIV-infected patients [28-31]. Our finding that the majority of patients responding to DPX-0907 generate such multi-functional T cells is encouraging and suggests that we may be generating clinically-relevant immune responses and that the immunogenic antigens have been combined with a suitably immunogenic delivery mechanism. DPX-0907 also includes a T helper epitope that has previously used to augment peptide vaccine immunogenicity [12,25]. Although the focus was to study antigen-specific CD8^+^ T cells, CD4^+^T cell responses werea lso detected against the A16L peptide in vaccinated patients, and these responses correlated with CD8^+^ T cell responses in the same assay. This suggests that the inclusion of a T helper peptide may have facilitated the generation of the specific CD8 T cells observed.

All the antigens contained in DPX-0907 are differentially presented by tumor cells [5] and have been validated experimentally [7,11]. Atleast three antigens (P4, P5 and P15, representing peptides from topoisomerase IIa (TOP2A), Integrin β8-precursor, and EDDR1 respectively) have been demonstrated to be upregulated at the mRNA level in ovarian cancer (unpublished observations). TOP2A (P4) is also associated with rapidly progressing cancers and relatively poor survival while EDDR1 (P15), a protein receptor kinase found to be over-expressed in ovarian cancers, is associated with poor prognosis and overall survival [8,32]. In addition, Tumor necrosis factor-Alpha Converting Enzyme (TACE/ADAM-17, P13) generates soluble epidermal growth factor receptor (EGFR) ligands such as EGF and HB-EGF that have been shown to be important in tumorigenesis [33].

In our study, we detected immune responses to 6 of the 7 immunizing antigens (P3, P4, P5, P7, P13 and P15) contained in DPX-0907. We saw the generation of responses to multiple antigens in several patients by MHC-pentamer and ICS assays and were able to confirm the observed responses by ELISpot in two responding patients. Our analysis also demonstrated pre-existing immune responses to a number of these antigens in our patient population, which suggests that patients’ T cells have been primed against these antigens prior to patient inclusion on this study. This provides further validation that the chosen vaccine epitopes are biologically relevant targets. The HLA-A2 restriction of the vaccine is expected to reduce eligibility of the Caucasian patient population by 50%, however future vaccines could incorporate peptides of other HLA restrictions or more promiscuous peptides to allow for broader utility of the vaccine. The multi-antigen approach is expected to minimize the impact of immune editing of one or more antigens by cancer cells under targeted immune pressure. The impact of possible HLA down-regulation in advanced cancer patients on the activity of this vaccine remains unknown.

Immune responses to DPX-0907 antigens were more frequent in breast and ovarian cancer patients as compared to prostate cancer patients. It is unclear whether this result indicates that ovarian and breast cancer patients are biologically more immune responsive than prostate cancer patients or whether this reflects other confounding clinical factors. We note however that most of the breast and ovarian cancer patients had stable disease or responses to their previous treatment, whereas the prostate cancer patients enrolled had progressive disease and increasing PSAs. In addition, all prostate cancer patients entered in this trial had greater than or equal to three previous treatments and demonstrated progression necessitating some other treatment soon after the completion of the three vaccinations. The observed differences between prostate cancer and other patients in this study were not statistically significant due to the small sample size; however, our results indicate that patients with a history of favorable responses to therapy, in a state of minimal residual disease or with fewer previous treatments are likely preferred candidates for treatment with DPX-0907, as has been proposed by others [34].

In summary, we have shown that this novel cancer vaccine – DPX-0907 – employing an innovative liposomal formulation is safe, immunogenic and worthy of further clinical testing. These studies will likely be required to optimize the patient populations treated and potentially incorporate an immune modulator to further optimize the antigen-specific immune responses. Future strategies will explore this and other antigen vaccines utilizing the DepoVax formulation, to generate functionally relevant clinical response able to reduce or eliminate micometastatic disease.

## Competing interests

NLB, MAM, JJN, GC, HK and KO received financial support from Immunovaccine to support the research described in the manuscript. MK, RN, LS, LDM, GMW, MMS and MM are employed by and/or stockholders of Immunovaccine.

## Authors’ contributions

NLB and MM participated in the design and coordination of the study, data acquisition and analysis and helped draft the manuscript, MK participated in the coordination of the study, data acquisition, immune data analysis and helped draft the manuscript, RN, MAM, JJN, GC, HK and KO participated in the design of the study, recruitment of patients and helped draft the manuscript, GMW, LS, LDM and MMS participated in the coordination of the study and helped draft the manuscript. All authors gave final approval of the manuscript for publication.

## Supplementary Material

Additional file 1 **Figure S1.** Clinical protocol outline and dosing schedule of DPX-0907 for the treatment of breast, ovarian and prostate cancer patients. Patients were pre-screened for determining their eligibility to participate in the study and were assigned to dose A or B of vaccine treatment as indicated. Blood samples were collected at pre-screening visit, during 3 treatments and at 1, 3 and 6 month post-treatment follow up. Immune monitoring was performed on PBMC from SD0, SD21, SD42 and SD73. **Figure S2**. Antigenic peptide-induced cytokine secretion by PBMC from DPX-0907 treated breast cancer patients. Patient PBMC were stimulated ex vivo for 6 h in the presence of pooled peptides included in DPX-0907, and protein transport inhibitor. Cells were surface stained for CD3, CD8, CD27 and CD45RA, permeabilized and stained for intracellular cytokines. Data represent percentage of total CD8^+^ T cells and/or central memory (T_CM_) CD8 T cells positive for cytokine secretion following peptide stimulation. **Figure S3**. Representative pentamer staining dot plots from two ovarian (02-09, 05-15) and one prostate cancer patient (01-18) showing increase in antigen-specific CD8^+^ T cells post-DPX-0907 treatment as compared to base line (SD0). Patient PBMC were stimulated with indicated peptide, in the presence of cytokines and stained with MHC-pentamer reagents. Cells were collected using a live gate and CD3^+^ cells were further separated to CD8^+^ T cells. These cells were plotted by CD45RA staining versus pentamer positive staining. Data on top left quadrant represent percentage of CD45RA^neg/low^ activated cells that were stained positive for pentamer reagent prepared using corresponding peptide shown for each patient. **Figure S4**. DPX-0907 vaccine induces multi-functional T cells capable of secreting multiple cytokines. Pre- and post- treatment PBMC samples from a representative breast (04-06) and ovarian (05-14) cancer patient were stimulated with peptide pool and analyzed by multi-parametric flow cytometry. Simultaneous determination of T cell phenotype (total, T_CM_) and type of cytokine secreted (IFN-γ/TNF-α/IL-2) was performed using FACS DiVa software (BD Bioscience) and multifunctional cytokine analysis was performed after stringent gating of each cytokine positive population and subsequent Boolean gating with FlowJo software. **Figure S5**. IFN-γ ELISpot responses in DPX-0907-treated breast and ovarian cancer patient PBMC. PBMC from selected breast and ovarian cancer patients and from non-vaccinated female healthy control subjects (HC-1 and HC-2) were used in ELISpot plates to stimulate with individual and pooled DPX-0907 peptides (10, 25, 50 and 100ug/ml tested and 50 μg/ml response shown) as described in the Materials and Methods. Mean ± SD SFU were plotted from the triplicate wells and expressed per 3x10^5^ cells that were plated per well. Click here for file

Additional file 2 **Table S1.** Tumor-associated antigens and corresponding peptides included in DPX-0907. **Table S2**. Patient Demographics. **Table S3.** Detection of antigen-specific CD8^+^ T cells in patients with immune responses to DPX-0907 treatment. Click here for file
