# Supplementary figures and images for: First-in-man application of a novel therapeutic cancer vaccine formulation with the capacity to induce multi-functional T cell responses in ovarian, breast and prostate cancer patients
Source: J Transl Med. 2012 Aug 3;10:156. doi: 10.1186/1479-5876-10-156 (PMC3479010; doi:10.1186/1479-5876-10-156)

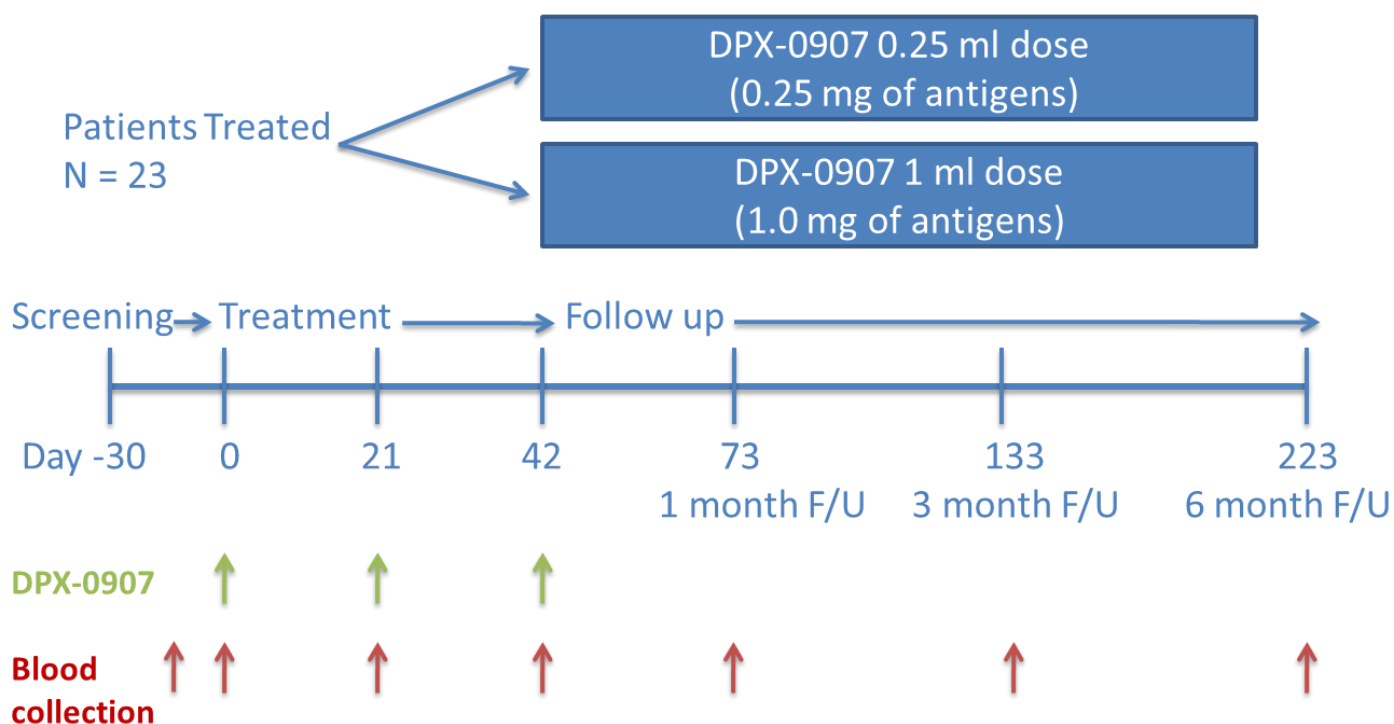

Supplemental Figure 1

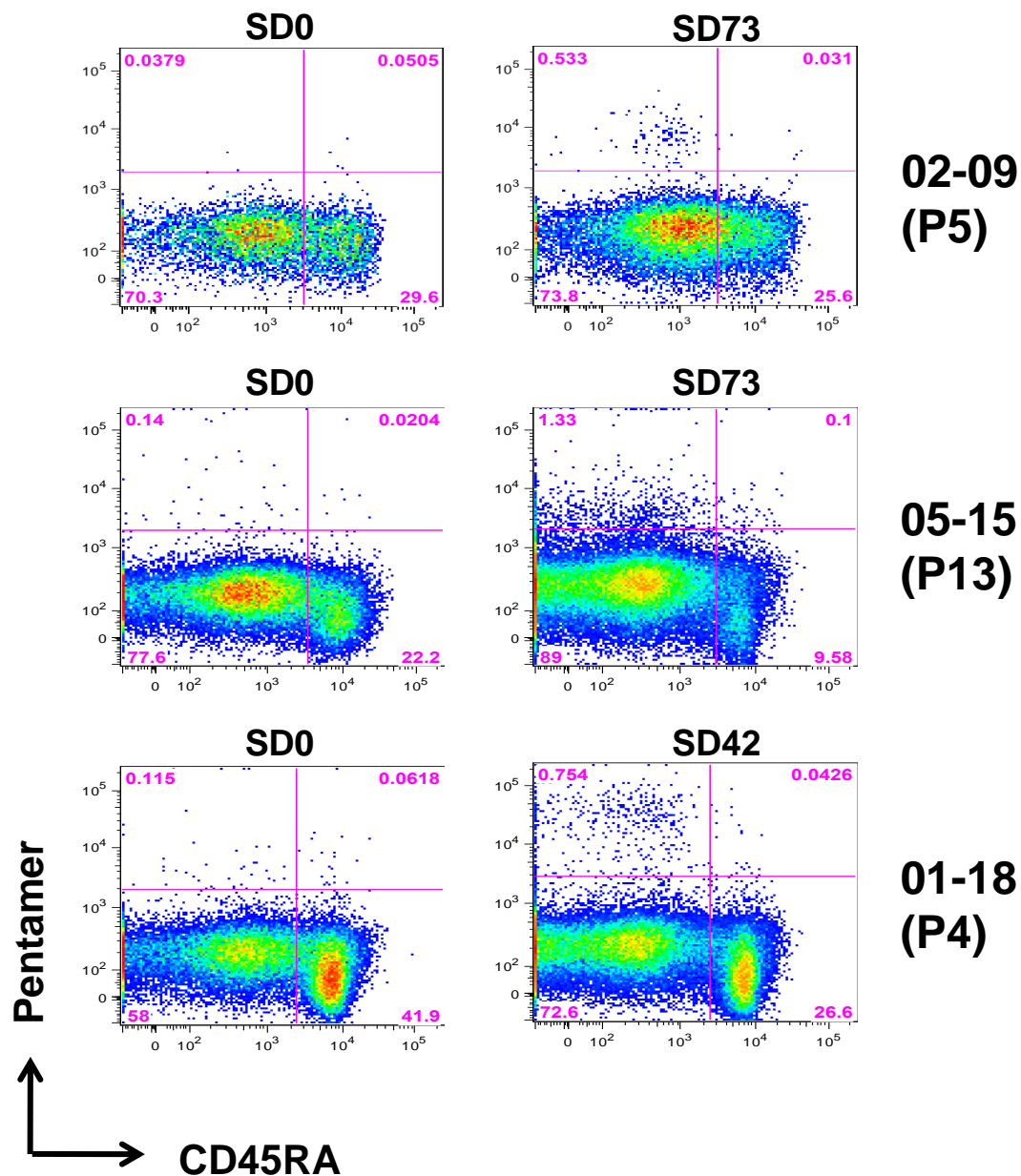

Supplemental Figure 2

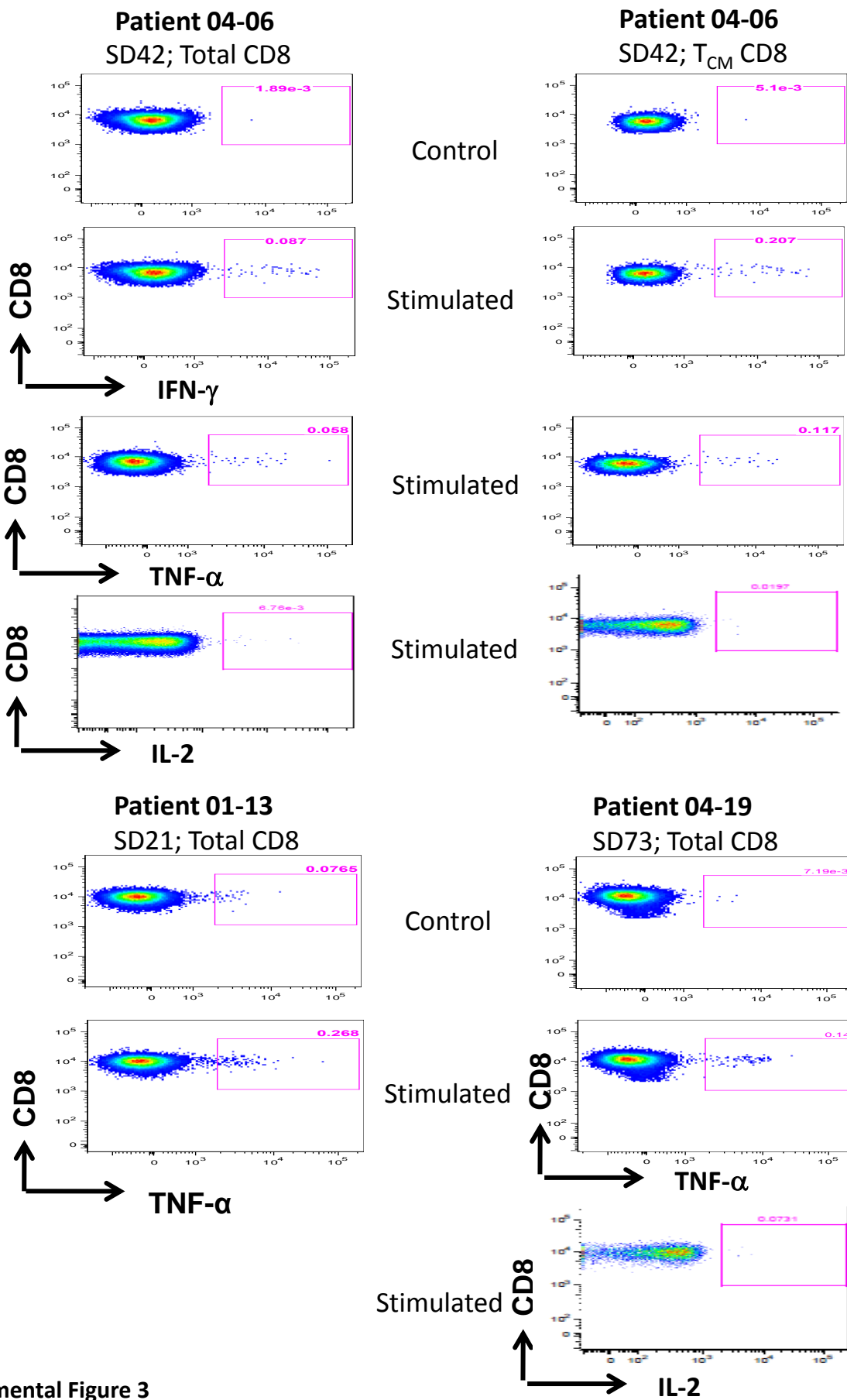

## Total CD8<sup>+</sup> T cells

## CD8<sup>+</sup> T<sub>CM</sub> T cells

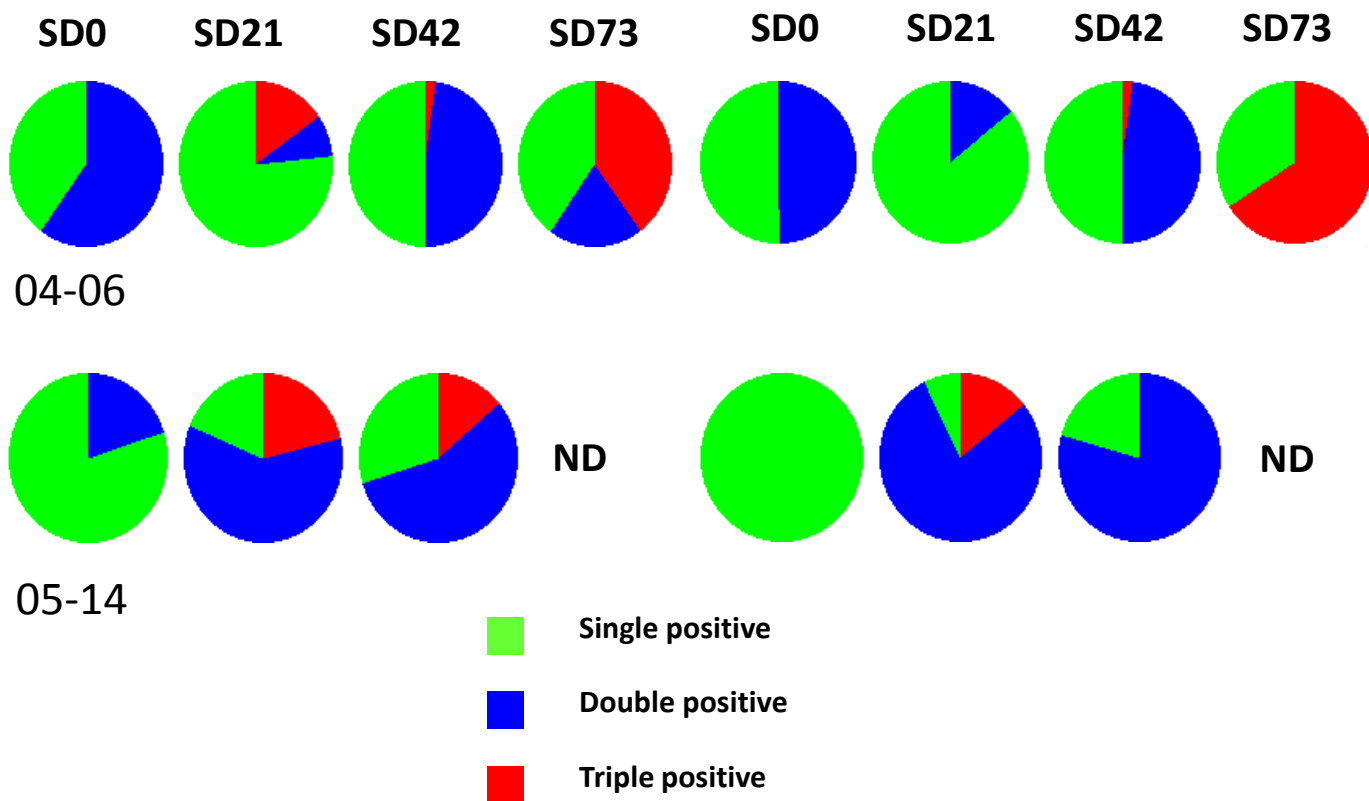

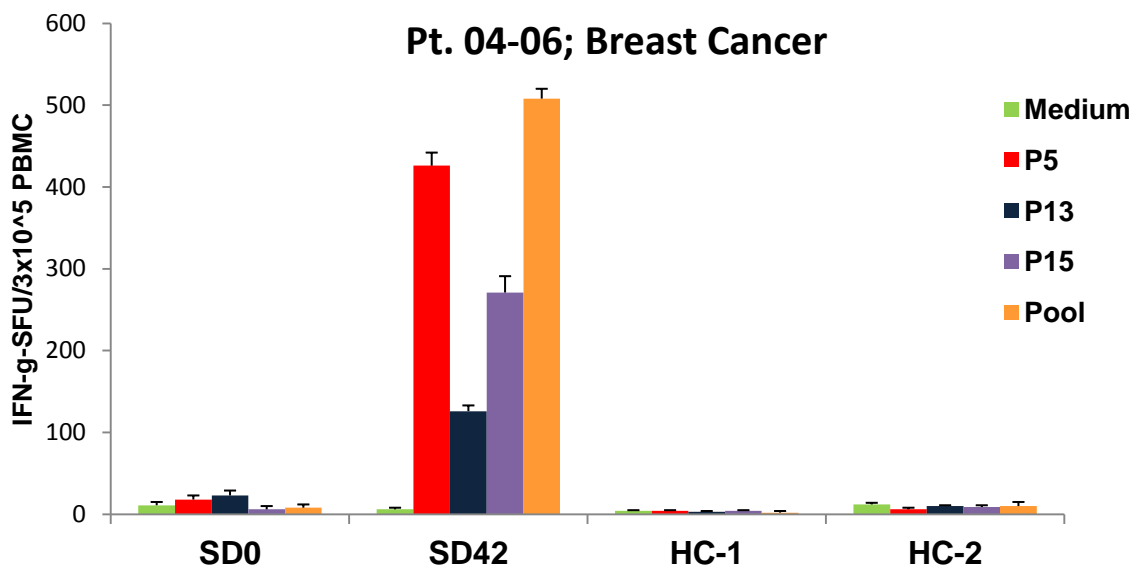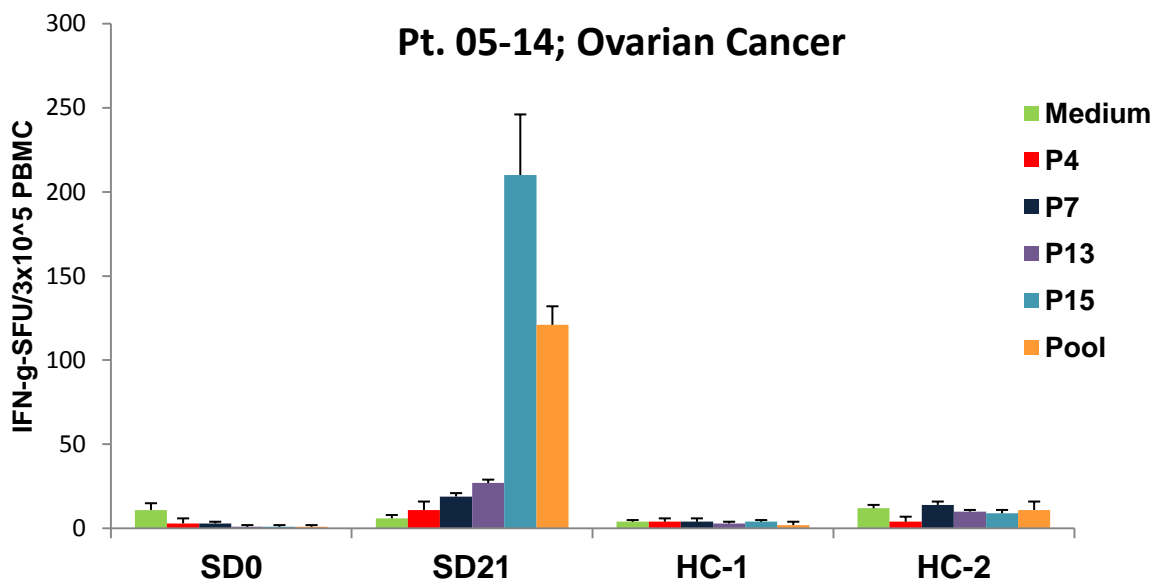

Supplement: Additional file 1 — Figure S1. Clinical protocol outline and dosing schedule of DPX-0907 for the treatment of breast, ovarian and prostate cancer patients. Patients were pre-screened for determining their eligibility to participate in the study and were assigned to dose A or B of vaccine treatment as indicated. Blood samples were collected at pre-screening visit, during 3 treatments and at 1, 3 and 6 month post-treatment follow up. Immune monitoring was performed on PBMC from SD0, SD21, SD42 and SD73. Figure S2. Antigenic peptide-induced cytokine secretion by PBMC from DPX-0907 treated breast cancer patients. Patient PBMC were stimulated ex vivo for 6 h in the presence of pooled peptides included in DPX-0907, and protein transport inhibitor. Cells were surface stained for CD3, CD8, CD27 and CD45RA, permeabilized and stained for intracellular cytokines. Data represent percentage of total CD8+ T cells and/or central memory (TCM) CD8 T cells positive for cytokine secretion following peptide stimulation. Figure S3. Representative pentamer staining dot plots from two ovarian (02-09, 05-15) and one prostate cancer patient (01-18) showing increase in antigen-specific CD8+ T cells post-DPX-0907 treatment as compared to base line (SD0). Patient PBMC were stimulated with indicated peptide, in the presence of cytokines and stained with MHC-pentamer reagents. Cells were collected using a live gate and CD3+ cells were further separated to CD8+ T cells. These cells were plotted by CD45RA staining versus pentamer positive staining. Data on top left quadrant represent percentage of CD45RAneg/low activated cells that were stained positive for pentamer reagent prepared using corresponding peptide shown for each patient. Figure S4. DPX-0907 vaccine induces multi-functional T cells capable of secreting multiple cytokines. Pre- and post- treatment PBMC samples from a representative breast (04-06) and ovarian (05-14) cancer patient were stimulated with peptide pool and analyzed by multi-parametric flow cyto [file 1479-5876-10-156-S1.pdf]
