# Supplementary material for: First-in-man application of a novel therapeutic cancer vaccine formulation with the capacity to induce multi-functional T cell responses in ovarian, breast and prostate cancer patients
Source: J Transl Med. 2012 Aug 3;10:156. doi: 10.1186/1479-5876-10-156 (PMC3479010; doi:10.1186/1479-5876-10-156)
Supplement: Additional file 2 — Table S1. Tumor-associated antigens and corresponding peptides included in DPX-0907. Table S2. Patient Demographics. Table S3. Detection of antigen-specific CD8+ T cells in patients with immune responses to DPX-0907 treatment. [file 1479-5876-10-156-S2.docx]

**Supplemental Figure Legends**

**Supplemental Figure 1**. Clinical protocol outline and dosing schedule of DPX-0907 for the treatment of breast, ovarian and prostate cancer patients. Patients were pre-screened for determining their eligibility to participate in the study and were assigned to dose A or B of vaccine treatment as indicated. Blood samples were collected at pre-screening visit, during 3 treatments and at 1, 3 and 6 month post-treatment follow up. Immune monitoring was performed on PBMC from SD0, SD21, SD42 and SD73.

**Supplemental Figure 2**. Representative pentamer staining dot plots from two ovarian (02-09, 05-15) and one prostate cancer patient (01-18) showing increase in antigen-specific CD8^+^ T cells post-DPX-0907 treatment as compared to base line (SD0). Patient PBMC were stimulated with indicated peptide, in the presence of cytokines and stained with MHC-pentamer reagents. Live gate was set up and CD3^+^ cells were further gated to include CD8^+^ T cells. Cells were plotted CD45RA staining versus pentamer positive staining. Data on top left quadrant represent percentage of CD45RA^neg/low^ activated cells that were stained positive for pentamer reagent prepared using corresponding peptide shown for each patient.

**Supplemental Figure 3**. Antigenic peptide-induced cytokine secretion by PBMC from DPX-0907 treated breast cancer patients. Patient PBMC were stimulated *ex vivo* for 6h in the presence of pooled peptides included in DPX-0907, and protein transport inhibitor. Cells were surface stained for CD3, CD8, CD27, and CD45RA, permeabilized and stained for intracellular cytokines. Data represent percentage of total CD8^+^ T cells and/or central memory (T_CM_) CD8 T cells positive for cytokine secretion following peptide stimulation.

**Supplemental Figure 4**. DPX-0907 vaccine induces poly-functional T cells capable of secreting multiple cytokines. Pre- and post- treatment PBMC samples from a representative breast (04-06) and ovarian (05-14) cancer patient were stimulated with peptide pool and analyzed by multi-parametric flow cytometry. Simultaneous determination of T cell phenotype (total, T_CM_) and type of cytokine secreted (IFN-γ/TNF-α/IL-2) was performed using FACS DiVa software (BD Bioscience) and multifunctional cytokine analysis was performed after stringent gating of each cytokine positive population and subsequent Boolean gating with FlowJo software.

**Supplemental Figure 5**. IFN-γ ELISpot response in DPX-0907-treated breast and ovarian cancer patient PBMC. PBMC from selected breast and ovarian cancer patients and from non-vaccinated female healthy control subjects (HC-1 and HC-2) were used in ELISpot plates to stimulate with individual and pooled DPX-0907 peptides (10, 25, 50 and 100ug/ml tested and 50 µg/ml response shown) as described in the Methods. Mean ± SD SFU were plotted from the triplicate wells and expressed per 3x10^5^ cells that were plated per well.
